# Functional Capacity Evaluation in Upper Limb Reduction Deficiency and Amputation: Development and Pilot Testing

**DOI:** 10.1007/s10926-017-9703-4

**Published:** 2017-04-10

**Authors:** S. G. Postema, R. M. Bongers, M. F. Reneman, C. K. van der Sluis

**Affiliations:** 1 Department of Rehabilitation Medicine, University Medical Center Groningen, University of Groningen, PO BOX 30001, 9700 RB Groningen, The Netherlands; 2 Center of Human Movement Sciences, University Medical Center Groningen, University of Groningen, Groningen, The Netherlands

**Keywords:** Occupational rehabilitation, Functional capacity evaluation, Upper limb amputation, Validity, Complaints of arms neck shoulder

## Abstract

*Purpose* To develop and pilot test a functional capacity evaluation (FCE) for individuals with upper limb absence (ULA) due to reduction deficiency or amputation, and to examine the relationship between FCE results and presence of musculoskeletal complaints (MSC). *Method* Five tests (overhead lifting, overhead working, repetitive reaching, fingertip dexterity, and handgrip strength) were selected and adapted if necessary. The newly developed FCE, called FCE-One-Handed (FCE-OH), was pilot tested in 20 adults individuals with ULA, and 20 matched controls. MSC were assessed via a questionnaire. *Results* Adaptations were considered necessary for all tests, except the handgrip strength test. The repetitive overhead lifting test of the non-affected limb was added. On the overhead lifting test, individuals with above-elbow ULA (ten males), performed similar to controls using one hand. When lifting bimanually using the prosthesis, a trend for lower lifting capacity of individuals with below-elbow ULA (seven males, three females) was observed compared to controls. On the overhead working test, individuals with above-elbow ULA performed worse compared to controls. Other tests showed no significant differences between groups. Relationships between FCE results and presence of MSC were non-significant. *Conclusion* The FCE-OH can be used to test functional capacity of one-handed individuals. Individuals with ULA generally showed similar functional capacity as two-handed individuals. FCE results were not related to MSC. It was discussed that a higher physical load on the non-affected limb might reflect a relative deficit of functional capacity.

## Introduction

Musculoskeletal complaints (MSC) appear to occur approximately twice as often in individuals with a congenital reduction deficiency or amputation of the upper limb [hereafter referred to as upper limb absence (ULA)] [1, 2]. Individuals with ULA are exposed to an unequal distribution of physical demands over both limbs, back and neck. The higher load on the non-affected limb, back and neck may result in overuse injuries of these sites. The number of repetitions, the magnitude of produced force and posture have been described extensively as risk factors for MSC in the general population [3–5], which may be increased in individuals with ULA. In addition, the higher burden on the non-affected limb may go along with a higher repetition of movements. Force and repetition have an interactive effect; a combination of high force and high repetition renders a high increase in MSC risk [6]. It was suggested that force requirements are increased when awkward or non-neutral positions are used [6], which may be the case when daily tasks are performed with a stiff wrist, such as with most prostheses [7–9]. Therefore, compensatory movements when wearing a prosthesis may possibly relate to overuse injuries of the affected limb.

Individuals with ULA may thus require higher functional capacity to perform daily tasks compared to two-handed individuals, because the daily physical load is mostly transferred to one limb only. Therefore, assessing functional capacity of individuals with ULA might be important for primary and secondary prevention of MSC. An instrument that assesses functional capacity may guide rehabilitation professionals to give appropriate advice to individuals with ULA regarding work participation, and should lead to less MSC. Furthermore, such an instrument may be used in rehabilitation programs to monitor functional capacity and measure the outcomes of the rehabilitation program.

In rehabilitation and occupational medicine functional capacity evaluations (FCE) are used to assess functional capacity. The term capacity test is used as a consistent terminology for all tests that measure the highest probable level of functioning that a person may reach in a domain at a given moment in a standardized environment [10]. An FCE is a comprehensive standardized assessment of an individual’s ability to perform work-related tasks [11].

Presently available FCE tests were developed for two-handed individuals and may not be suited to assess functional capacity of one-handed individuals. This study was conducted to adapt existing FCE tests for use in individuals with ULA with and without prosthesis. Further aims of this study were to pilot test the adapted FCE in individuals with ULA, and compare test results with the functional capacity of matched controls. Because functional capacity of individuals with ULA has not been researched previously, we proposed two competing hypotheses. First, we hypothesize a higher functional capacity of individuals with ULA as a consequence of a proper adaption to the higher load on the non-affected limb. On the other hand, individuals may fail to adapt to the redistributed functional loads, and are consequently prone to early onset of muscle fatigue or MSC. Our second hypothesis contains a lower functional capacity for individuals with ULA. Furthermore, we examined the relationship between test results of individuals with ULA and presence of MSC.

## Method

FCE tests were selected and adapted for use in individuals with an upper limb amputation, which is described in part one of the method. Part two describes the participants and test-procedure. This study was approved by the local ethical committee (NL43394.042.13) and each participant gave informed consent.

### Part 1: Selection of FCE Tests

FCE test selection was based on a study on physical risk factors for work related upper limb disorders [12]. These tests were extracted from the Work Well FCE [11]. Safety, reliability, validity, and reference values of most tests were established in healthy workers [13–17]. The selected tests, including a description of the original tests and the adaptations made, are described in Table 1. Test materials are described in detail elsewhere [12]. The overhead lifting test measures functional strength of shoulder and arm musculature, whereas the overhead working test measures the static holding time of shoulder and neck musculature, as well as endurance. These two tests are generally performed bimanually, but can be performed one-handed. The repetitive reaching test measures both endurance and coordination. The fingertip dexterity test and the hand grip strength test assess fine motor capacity of the fingers and isometric grip strength, respectively.

**Table 1:** Description of original and adapted FCE tests

Test (ICF code) (materials)	Description of original test	Adaptations	Extra test instructions		Measurement outcomes	Number of times the test is performed
			AE-ULA participants	BE-ULA participants		
Overhead lifting test (d4300)	Five lifts with a plastic receptacle from table to crown height and v.v. in standing position within 90 s. Weight increments 2 kg (females) or 4 kg (males) until maximum amount of kgs is reached If five lifts with a certain amount of kgs are not reached, a decrement of 1 kg (females) or 2 kg (males) may be tried	Fixed grasp positions	Performed one-handed. The unaffected hand grasps the front side of the receptacle	The non-affected hand and the prosthesis hand hold the handles of the receptacle	Maximum amount of kg that can be lifted five times from table to crown height, within 90 s	Once
Repetitive Overhead lifting test (one-handed)	*Not existing* Added to test the one-handed overhead lifting capacity	N/A	20 lifts with a weight of 1.9 kg from table to crown height and v.v. in standing position with the unaffected hand		Time (s) needed for 20 lifts from table to crown height, up and down counts as one	Once
Overhead working test (d4158)	Standing with hands at crown height, manipulating nuts and bolts, and wearing cuff weights of 1 kg around the forearms	No cuff around the prosthetic wrist	Performed one-handed	The prosthesis hand is used to fixate the nuts	Time (s) which the position can be held	Once
Repetitive reaching test (d4452)	Sitting with bowls on wingspan distance, move 30 marbles horizontally at table height from right to left and v.v. as fast as possible The test is performed with one arm	The bowls with marbles are replaced for a one-button clicking system. The procedure remains the same	Touch the button on the clicking system Performed with the unaffected hand only	Touch the button on the clicking system The test is performed with the unaffected hand and the prosthesis hand	Time (s) needed to make 30 reaching movements, back and forward counting as one	Three times; AE-ULA participants and controls: all trials with non-affected/dominant hand. BE-ULA participants and controls: first and third trial with non-affected/dominant hand, second trial with prosthesis hand/ braced non-dominant hand
Fingertip dexterity test (d4458) (Purdue pegboard, model 32020, J.A. Preston Corporation, New York, NY, USA)	Sitting in front of a Purdue pegboard, placing pins with left or right hand as fast as possible in a 30 s trial	When testing with the prosthesis: pins are picked up with the non-affected hand, transferred to the prosthesis and then placed in the pegboard	Performed with the unaffected hand only	When performed with the prosthesis hand: pins are picked up with the unaffected hand and manually placed in the prosthesis hand	Number of pins placed in 30 s	Three times; AE-ULA participants and controls: all trials with non-affected/dominant hand. BE-ULA participants and controls: first and third trial with non-affected/dominant hand, second trial with prosthesis hand/ braced non-dominant hand
Handgrip strength (d4400/s73022) (Hydraulic hand dynamometer, JAMAR, Sammons Preston Inc. Bollingbrook, IL, USA)	Sitting with the shoulder adducted and neutrally rotated, elbow flexed at approx. 90°, and the forearm and wrist in neutral position, pinching a hydraulic handgrip dynamometer (in position two of the five available positions [32]) for approx. 3 s	No adaptations	Performed with the unaffected hand only		Pinched kg	Three times with non-affected/dominant hand

Individuals with AE-ULA perform all tests one-handed, while individuals with BE-ULA use their prosthesis. Any type of below-elbow prosthesis (cosmetic, body-powered, or myoelectric) can be used for all tests, with the exception of the fingertip dexterity test, which cannot be performed with a cosmetic prosthesis

### Part 2: Measurements

#### Participants

During a previous survey among individuals with an upper limb reduction deficiency or amputation, participants were asked whether they were interested in information regarding follow-up research. Those who were interested, and lived on reasonable distance from the hospital, were sent written information regarding this study. Inclusion criteria were: being between 18 and 67 years of age, good comprehension of the Dutch or English language, and having ULA at or proximal to the carpal level. In case of a below-elbow amputation, the one-handed individual needed to own a well-fitting prosthesis (self-assessed by the participant; reasonable and good fitting were accepted for participation), and have experience using it. In addition, the participant’s blood pressure had to be below 159 mmHg (systolic) and 100 mmHg (diastolic). Exclusion criteria were: abnormal function of the unaffected hand (based on the participant’s own judgment), invalidating or serious pulmonary or cardiac health problems or other comorbidity that may influence the FCE test results.

Each participant with ULA was matched with a control person, based on gender, age (±5 years), weight (±10 kg) and height (±10 cm). Information flyers, placed in communal areas of the hospital, were used to recruit controls. In addition, controls were recruited among acquaintances of the researchers. The same inclusion and exclusion criteria were used, with exception of the presence of ULA. Both hands needed to have normal function, which was determined by the control’s own judgment.

#### Test Procedure

Before starting with the measurements participants answered the 7-item Physical Activity Readiness Questionnaire (PAR-Q) [18], in order to assess the exclusion criteria. The PAR-Q has been successfully used in FCE research before [17, 19]. If any item was answered positively, the participant was excluded from participation. Furthermore, the participants’ blood pressure was measured. After inclusion, the participant was instructed about the termination criteria during the procedure: (1) if the participant wanted to stop (termination induced by participant); (2) if the heart rate was above 85% of the age related maximum [(220 − age) × 85%] (termination induced by test leader); (3) loss of control, leading to an unsafe procedure (termination induced by test leader).

The tests were performed in random order. The controls performed the tests in the same order as the matched participant with ULA. A heart rate monitor (Polar Accurex Plus, Polar Electro Nederland BV, Almere, The Netherlands) was used to register heart rate before and after each test. Increase in heart rate was calculated as heart rate after the test divided by the heart rate before the test multiplied by 100%. Furthermore, after each test the CR10-Borg-scale was administered [20]. This 11-point Likert scale informs after the perceived exertion, and ranges from ‘rest’ (0) tot ‘maximum’ (10).

The six adapted FCE tests were performed, as described in Table 1. Participants with transradial ULA or wrist disarticulation [hereafter referred to as below-elbow ULA (BE-ULA)] used their prosthesis during the tests, while participants with a transhumeral ULA [hereafter referred to as above-elbow ULA (AE-ULA)] did not use a prosthesis (as several tests could not, or with much difficulty, be performed with an above-elbow prosthesis). Participants with BE-ULA were allowed to manually rotate the wrist of the prosthesis, before starting a test. Controls used a brace around their wrist, blocking wrist flexion/extension, ulnar/radial deviation, and pro- and supination, for those tests that were performed with a prosthesis by their match (Table 1). Furthermore, controls performed the overhead lifting test twice: once two-handed (normal condition), and once in an adapted condition (one-handed, or with a brace, depending on whether they were matched to an individual with AE-ULA or BE-ULA, respectively). Half of the controls performed the test first in the normal condition, and the other half in the adapted condition. The first controls were asked to perform the overhead working test twice too, once two-handed, and once in the adapted condition. However, soon an effect of fatigue was detected, as the second test was always performed worst, independent of the condition of this test, after which it was decided to let the remaining controls perform the test only in the normal condition.

After the measurements all participants filled out a questionnaire regarding personal characteristics and presence of MSC. Type of occupation was classified by the authors (SGP and MFR) into four categories: no employment, sedentary or light physical work demands, medium physical work demands, and heavy or very heavy physical work demands. Classification was based on the Dictionary of Occupational Titles [21]. Furthermore, the questionnaire informed after presence of MSC during the previous four weeks, the location and duration of complaints, the severity of the pain [on a 11-point Numeric Rating Scale (NRS) from 0 (no pain) to 10 (worst imaginable pain)], and the disability caused by the complaints. The latter was assessed with the Pain Disability Index (PDI) questionnaire [22, 23], assessing disability in seven domains in daily life (family and household, recreation, social activities, work, sexuality, self-care, and basic needs). Disability in each domain was scored on an NRS ranging from 0 (no disability) to 10 (worst disability), and answered were summed. Therefore, scores ranged from 0 to 70; with a higher score indicating greater disability.

### (Statistical) Analyses

Due to the relatively low number of participants in each group it was decided to perform all statistical analyses with non-parametric tests. For comparison of four groups (AE-ULA, BE-ULA, and two groups of matched controls) the independent sample Kruskal–Wallis test with a pairwise comparison was used. The test statistic (H-statistic), degrees of freedom (df) and effect sizes of significant pairwise comparisons are given. For comparison of two groups the unpaired sample Mann–Whitney *U* test was used. The test statistic (U-statistic) and effect size are presented. Furthermore, for comparison of the dominant limb and the prosthetic limb within the group of BE-ULA the related sample Wilcoxon signed rank test was used. Also for this test the test statistic (T-statistic) and effect size are given. Effect sizes are presented as the Pearson’s correlation coefficient *r*, calculated by dividing the *z*-value (standardized test statistic) by the root of the number of observations. *r* = .10 is interpreted as a small effect, *r* = .30 as a medium effect, and *r* = .50 as a large effect [24]. If for any reason an individual was excluded from analyses, his or her match was also excluded from those analyses. Results are presented as median (IQR), and *p* values of ≤0.05 were considered statistically significant.

## Results

### Part 1: Adaptation of FCE Tests

The researchers, who have experience in the fields of FCE, upper limb reduction deficiency and amputation rehabilitation care or movement sciences, discussed the original tests to decide which adaptations were necessary so that the tests could be performed by one-handed individuals with or without a prosthesis, and would measure functional capacity rather than prosthesis handling skills. Discussions were held until consensus between all researchers was reached (Table 1). As these tests are adapted for use in one-handed individuals, we propose the name Functional Capacity Tests-One-Handed (FCE-OH).

### Part 2: Measurement Results

The total number of participants was 40, divided over four groups (Table 2).

**Table 2:** Description of the studied participants

	Participant groups				Test statistic (df)	*p*
	AE-ULA	AE-C	BE-ULA	BE-C		
Male/female ratio (n)	10/0	10/0	7/3	7/3		
Age (years) [median (IQR)]	49.0 (39.2; 52.9)	54.3 (34.4; 58.3)	48.5 (39.2; 51.0)	45.9 (34.0; 55.4)	1.3 (3)	0.724
Height (cm) [median (IQR)]	179.0 (173.8; 188.6)	183.5 (175.9; 186.3)	181.8 (170.6; 184.8)	183.0 (169.1; 189.9)	0.6 (3)	0.894
Weight (kg) [median (IQR)]	82.2 (76.5; 91.7)	86.6 (80.5; 94.3)	87.1 (82.6; 98.3)	86.7 (80.3; 95.9)	1.5 (3)	0.678
Occupation (n)						
Not employed	4	0	3	2		
Sedentary or light	5	6	7	7		
Medium	0	4	0	1		
Heavy or very heavy	1	0	0	0		
Cause of ULA (n)						
Congenital	0	N/A	7	N/A		
Trauma	10		3			
Type of prosthesis (n)						
Cosmetic	^a^	N/A	1	N/A		
Body-powered			2			
Myoelectric			7^b^			
Prosthesis use (n)						
Sometimes	^a^	N/A	1	N/A		
Always			9			
Prosthesis fitting of socket (n)						
Reasonable	^a^	N/A	1	N/A		
Good			9			
Dominant hand (n)^c^						
Left	5	2	4	2		
Right	5	7	6	8		
Ambidextrous	–	1	–	0		

*C* controls, *N*/*A* not applicable

^a^Participants with an AE-ULA did not use their prosthesis during the measurements. Therefore no details about their prosthesis and prosthesis use are provided here. Five of the ten participants did use a prosthesis in daily life

^b^One of the participants with a myoelectric prosthesis had a multi-articulating prosthesis hand

^c^The dominant hand is stated; for the participants with ULA this is the non-affected limb. Therefore five individuals with AE-ULA and six individuals with BE-ULA had a deficiency or amputation of the left limb

#### FCE-OH Test Results: Differences Between Individuals with ULA and Controls

Results of the FCE-OH tests are described in Table 3. Participants with AE-ULA lifted similar weight as their matched controls, when the latter lifted with one hand. However, when the matched controls lifted with two hands, the individuals with AE-ULA lifted significantly less weight. For individuals with BE-ULA, reasons for not adding another increment of weight during the overhead lifting test were bad grip with the prosthesis hand, leading to unsafe lifting (n = 6), reaching maximal lifting capacity (n = 3), and increasing pain in the non-affected hand (n = 1). Individuals with an above-elbow amputation lifted the receptacle with one hand, and mentioned reaching maximal lifting capacity and unsafety equally often as reason to stop (both: n = 5). Reaching maximal lifting capacity was the most called reason to stop for most controls (n = 15). Other reasons were unsafety (n = 4) and fear for physical harm (n = 1).

**Table 3:** Results of individuals with ULA and controls on adapted FCE tests, increase in heart rate while performing these tests and perceived exertion of the tests, measured with the Borg scale

	AE-ULA	AE-C	BE-ULA	BE-C	Test statistic (df)	p*	Post hoc comparison^e^	Effect size	Missing values^f^
Test results									
Overhead lifting test^a^	8.0 (5.5; 10.0)	20.0 (20.0; 24.0)	11.0 (7.3; 16.0)	20.0 (15.0; 24.5)	24.8 (3)	<0.001	AE-ULA and AE-C: <0.001	−0.97	0/60
Overhead lifting test^b^	8.0 (5.5; 10.0)	8.0 (8.0; 9.0)	11.0 (7.3; 16.0)	19.0 (10.8; 22.5)	10.3 (3)	0.016			0/60
Repetitive overhead lifting test—one-handed	41.0 (37.8; 47.8)	40.5 (34.0; 46.3)	38.5 (36.0; 41.3)	36.5 (33.3; 42.3)	3.5 (3)	0.320			0/40
Overhead working test^c^	203.5 (133.0; 263.0)	313.5 (238.0; 603.0)	250.5 (182.8; 339.8)	256.0 (182.8; 353.8)	8.5 (3)	0.037	AE-ULA and AE-C: 0.023	0.65	0/40
Overhead working test^d^	158.0 (118.0; 244.0)	325.0 (300.0; 521.0)	230.0 (169.5; 360.5)	243.0 (168.5; 381.5)	9.0 (3)	0.030	AE-ULA and AE-C: 0.018	0.79	4/40 (8)
Repetitive reaching test—with dominant hand	58.7 (53.6; 59.8)	58.3 (48.7; 65.2)	54.5 (51.9; 58.5)	55.3 (51.8; 63.6)	1.2 (3)	0.744			0/40
Repetitive reaching test—with prosthesis/brace	N/A	N/A	67.5 (56.8; 77.3)	65.0 (59.3; 78.3)	55.0	0.739		0.08	0/40
Fingertip dexterity test—with dominant hand	12.7 (11.3; 14.4)	14.3 (13.0; 14.8)	15.5 (13.9; 17.4)	14.0 (12.9; 18.1)	6.4 (3)	0.096			0/40
Fingertip dexterity test—with prosthesis/brace	N/A	N/A	6.5 (5.0; 8.0)	4.1 (1.3; 6.8)	17.5	0.130		−0.38	2/20 (4)
Hand grip strength—with dominant hand	40.3 (34.8; 47.8)	47.7 (40.3; 51.8)	38.3 (31.1; 51.1)	37.5 (34.9; 42.5)	3.1 (3)	0.371			0/40
Heart rate results									
Overhead lifting test^a^	114.6 (109.7; 116.4)	142.0 (139.4; 162.2)	138.8 (121.8; 144.3)	138.6 (129.5; 156.8)	17.2 (3)	0.001	AE-ULA and AE-C: <0.001	−0.94	4/40 (8)
Overhead lifting test^b^	114.6 (109.5; 116.5)	119.3 (111.1; 139.6)	138.8 (121.8; 150.6)	144.1 (131.7; 156.8)	11.3 (3)	0.010			5/40 (10)
Repetitive overhead lifting test—one-handed	116.4 (109.4; 121.7)	112.8 (106.7; 125.4)	114.3 (108.0; 120.7)	123.8 (113.5; 130.0)	2.8 (3)	0.425			0/40
Overhead working test^c^	106.8 (101.8; 111.6)	115.1 (109.8; 120.5)	106.7 (103.5; 121.8)	114.0 (108.6; 117.4)	4.3 (3)	0.230			1/40 (2)
Overhead working test^d^	106.1 (97.8; 115.8)	117.5 (114.0; 123.2)	105.8 (103.4; 117.5)	115.3 (108.1; 117.4)	5.5 (3)	0.137			1/32 (2)
Repetitive reaching test—with dominant hand	125.2 (113.1; 135.7)	129.0 (121.9; 141.8)	121.9 (111.6; 135.0)	129.1 (120.5; 141.3)	3.4 (3)	0.334			1/40 (2)
Repetitive reaching test—with prosthesis/brace	N/A	N/A	118.3 (114.8; 126.4)	132.9 (118.1; 143.0)	68.0	0.190		0.30	0/16
Borg scale results									
Overhead lifting test^a^	5.0 (4.0; 5.0)	7.0 (6.5; 7.3)	5.0 (4.0; 7.0)	6.0 (4.0; 8.0)	8.3 (3)	0.040	AE-ULA and AE-C: 0.027*	−0.64	1/40 (2)
Overhead lifting test^b^	5.0 (4.0; 5.0)	5.0 (4.0; 5.8)	5.0 (4.0; 7.0)	6.0 (4.5; 7.5)	2.9 (3)	0.406			1/40 (2)
Repetitive overhead lifting test—one-handed	2.0 (1.8; 2.3)	2.0 (1.0; 2.3)	2.0 (1.0; 3.3)	2.0 (1.0; 3.0)	0.5 (3)	0.921			0/40
Overhead working test^c^	4.0 (3.0; 5.0)	4.5 (3.8; 6.3)	4.5 (2.8; 5.0)	5.0 (3.0; 6.0)	1.9 (3)	0.584			0/40
Overhead working test^d^	4.0 (3.0; 5.0)	5.0 (4.0; 7.0)	4.0 (2.5; 5.0)	5.0 (3.5; 6.0)	3.8 (3)	0.288			0/32
Repetitive reaching test—with dominant hand	2.0 (1.0; 2.5)	1.2 (0.0; 2.1)	2.8 (1.8; 3.0)	2.8 (2.0; 3.1)	10.0 (3)	0.019			0/40
Repetitive reaching test—with prosthesis/brace	N/A	N/A	3.0 (1.8; 4.0)	3.0 (2.0; 4.0)	54.0	0.796		0.07	0/40
Fingertip dexterity test—with dominant hand	0.0 (0.0; 1.3)	0.0 (0.0; 0.3)	0.0 (0.0; 0.9)	0.3 (0.0; 1.0)	6.4 (3)	0.096			0/40
Fingertip dexterity test—with prosthesis/brace	N/A	N/A	1.0 (0.0; 3.0)	1.0 (0.0; 1.0)	27.0	0.645		−0.14	0/16

Data is presented as median (IQR)

*C* controls, *N*/*A* not applicable

*p values compare the four groups (AE-ULA, AE-C, BE-ULA, and BE-C) with an independent sample Kruskall-Wallis test with a pairwise comparison (next column)

^a^Controls performed the test in the original condition, that is, two-handed

^b^Controls performed the test in an adapted condition, that is one-handed (AE-C) or with a brace (BE-C)

^c^All participants included

^d^Four participants and their matches excluded, as they performed the test first in adapted condition (one-handed, or with brace)

^e^Only significant post hoc comparisons between matched groups are presented

^f^The number of missing values are given per number of observations; between brackets the number of excluded observations are given. Two individuals with BE-ULA, as well as their matched controls, were excluded from analyses of the fingertip dexterity test with the prosthesis, as they could not perform the test with their prosthesis. In eight cases (n = 6 for the overhead lifting test, n = 1 for the overhead working test, and n = 1 for the repetitive reaching test with the dominant hand) the heart rate was not asked or asked too late, therefore these observations, including the observations of their matches, were excluded from analyses

Participants with AE-ULA performed worse on the overhead working test compared to their matched controls.

#### Learning Effects for Repetitive Reaching Test and Fingertip Dexterity Test

Of all 40 individuals, 38 performed the repetitive reaching test the third trial faster compared to the first trial. Differences in time between the first and third trial ranged between −5 and 17 s, with a median of 7.0 (IQR 5.0; 9.0). This effect of practicing was also present in the fingertip dexterity test, where 26 out of 40 scored better on the third trial compared to the first trial [median difference: 1.5 (IQR 0.0; 3.0)].

#### Comparison of the Non-affected and Prosthetic Limb

Prosthesis users performed the repetitive reaching test faster with their non-affected hand compared to their prosthetic hand [54.4 s (IQR 51.9; 58.5) and 67.5 s (IQR 56.8; 77.3), respectively, T = 49.5, *p* = .025, *r* = .50]. Two individuals did not perform the fingertip dexterity test with the prosthesis, as one individual had a cosmetic prosthesis, and the myoelectric prosthesis of the second did not function well. The eight individuals who performed the fingertip dexterity test both with the non-affected hand and the prosthesis, performed the test better with the non-affected hand [15.5 pins (IQR 13.8; 18.1) and 6.5 pins (IQR 5.0; 8.0), respectively, T = 0.0, *p* = .012, *r* = −0.63].

#### Heart Rate and Perceived Exertion

During the overhead lifting test, individuals with AE-ULA showed a significant lower increase in heart rate compared to their matched controls when lifting two-handed, as well as a lower perceived exertion (Table 3).

#### Relationship Between Test Results and MSC

Nine out of the 20 participants with ULA experienced MSC in the previous four weeks: six men with AE-ULA, and two men and one woman with BE-ULA [median age 50.3 years (IQR 37.9; 52.6), four not employed, four with a sedentary or light occupation, and one with a heavy or very heavy occupation]. The complaints were located in the low back (n = 2), neck or high back (n = 4), shoulder of the non-affected limb (n = 6), the non-affected limb (shoulder not included) (n = 8), and the shoulder of the affected limb (n = 1). Seven individuals (five with AE-ULA and with BE-ULA) had complaints in more than one bodily location. Furthermore, six individuals (four with AE-ULA and two with BE-ULA) had chronic complaints (e.g. lasting more than 3 months). The median NRS-score for the pain was 3.5 (IQR 2.3; 6.5) (one missing value) and their mean PDI-score was 28.0 (IQR 10.5; 36.8) (one missing value). Differences in test results between individuals with and without MSC during the previous 4 weeks were not significant (Table 4).

**Table 4:** Relationship between FCE-OH tests and MSC in individuals with ULA

	Individuals with MSC (n = 9)	Individuals without MSC (n = 11)	Test statistic	*p*	Effect size
Overhead lifting test	8.0 (4.0; 10.0)	10.0 (8.0; 16.0)	24.0	0.056	0.44
Repetitive overhead lifting test—one-handed	40.0 (36.5; 45.0)	39.0 (38.0; 42.0)	48.5	0.941	−0.02
Overhead working test	219.0 (140.5; 306.0)	230.0 (188.0; 281.0)	49.5	1.000	0
Repetitive reaching test—with dominant hand	56.5 (53.4; 59.5)	54.5 (51.3; 59.3)	0.8	0.824	0.06
Fingertip dexterity test—with dominant hand	13.7 (12.3; 15.3)	14.5 (11.3; 16.0)	43.0	0.656	−0.11
Hand grip strength—with dominant hand	38.7 (33.7; 42.2)	45.3 (33.0; 51.3)	39.0	0.456	−0.18

Data is presented as median (IQR)

## Discussion

### Development and Pilot Testing of the FCE-OH

Existing FCE tests were adapted to develop an FCE for individuals with unilateral ULA, which was called FCE One-Handed. The FCE-OH consists of six tests, examining the functional strength, static holding time, endurance, and coordination of shoulder and arm musculature, fine motor capacity of the fingers, and isometric hand grip strength.

The adapted tests allow for assessment of functional capacity of individuals with one hand. Not all individuals with ULA use a prosthesis, and therefore it was decided to adapt the tests in a way that they can be performed both with and without a prosthesis. This makes the test also usable for patient groups that have one non-functional hand due to other conditions. While most tests are performed with the non-affected limb only, or with the non-affected limb and the prosthetic limb separately, it is possible to perform the overhead lifting test “bimanually”, that is, with the non-affected limb and the prosthetic limb (independent of the type of prosthesis) simultaneously. As the use of a prosthesis might influence the measured functional capacity of the upper limb musculature, no other “bimanual” tests were included.

Based on this pilot test, a few recommendations for future research and clinical use of this outcome measure can be made. First, due to alterations of the test, the repetitive reaching test with the clicking systems should not be compared with reference data of the original test with marbles. Obviously, picking up a marble costs more time than clicking a button. Results of other tests with minor alterations could be compared with reference data. Second, when administering the FCE-OH one should recognize the learning effect in the repetitive reaching test, and to a lesser extent also in the fingertip dexterity test. Last, several participants with ULA mentioned during the overhead lifting test that they had preferred to lift the container in another way, e.g. without the prosthesis, but using their stump. Therefore, the standardized test might not represent the true lifting capacity of individuals with ULA, and to assess work capacity of an individual, it is recommended to also test lifting capacity in the participant’s preferred manner.

### Functional Capacity of Individuals with ULA

Overhead lifting test is lower in one-handed individuals compared to controls. In individuals with BE-ULA there was a trend for a lower lifting capacity compared, suggesting that bimanual lifting capacity with a non-affected limb plus a prosthesis is lower than the lifting capacity of two non-affected limbs. Interestingly, the individuals with BE-ULA and their matched controls reported similar perceived exertion, indicating that, although less weight was lifted, the test was perceived equally physically demanding. The lifting capacity of the individuals with ULA (AE or BE) was low compared to the two-handed lifting capacity of the control group, and also compared to reference data of healthy Dutch adults [16]. However, it does meet the lifting criterion for light physical demanding occupations [16]. The functional capacity, as measured in this study, of the participants with ULA seems to match their work load. The test results of this study suggest that individuals with ULA generally could perform work with sedentary or light physical demands. However, suitability should be assessed on an individual level, in which the individual’s capacities and work demands are compared.

The most mentioned argument by individuals with BE-ULA for not increasing the lifted weight was bad grip with the prosthesis, leading to unsafe lifting. Thus, termination of the test was often not related to reaching maximal capacity of muscle strength. Ostlie et al. found that the strength of the shoulder muscles of the affected limb was not decreased in the majority (83–96%) of 66 unilateral upper limb amputees [25]. Apparently, more confidence regarding the prosthesis may increase lifting capacity. Possibly, prostheses with feedback, through for example osseointegration or pressure sensors, may allow individuals with ULA to rely more on the prosthesis, and thus increase the weight that can be lifted.

### Expectations and Results: Towards New Hypotheses

According to the dose–response model, exposure to physical exertions results in an “internal dose”, such as tissue loads and metabolic demands. These internal doses cause mechanical, physiological and psychological disturbances [26]. Repeated and sustained exertions can result in changes in tissue composition, which in turn may result in increased dose tolerance. This is referred to as adaptation, and is a desirable effect of training. The first of our two competing hypotheses stipulated that individuals with ULA adapt well to the internal disturbances that occur as a result of increased demands of the non-affected limb, and thus show a higher functional capacity. However, according to the dose–response model, adaptation to internal changes can also be compromised, reducing functional capacity. The musculoskeletal tissues may have insufficient capacity to repair sustained damages, resulting in muscle fatigue, MSC, and even long-lasting impairment. As a result, our second hypothesis stipulated that individuals with ULA show lower functional capacity.

The results from our study point more towards the second hypothesis than towards the first. Although absolute capacity of the upper limb musculature seems to be similar for one-handed and two-handed individuals, the misbalance in the burden on both limbs results in a higher load on the non-affected limb. Seen from this perspective, the similar capacity as observed may be interpreted as a relative deficit of capacity of the non-affected limb, because the demands on this limb are higher. We observed lower endurance when performing the overhead working test for individuals with AE-ULA compared to their matched controls. A compromised adaptation to the increased physical load may have resulted in overloaded muscle fibers of the non-affected limb. The Cinderella theory stipulates that sustained low-level isometric contractions set up a stereotyped recruitment pattern of type 1 motor units in the muscle, which are constantly active, even in situations where the total muscle load is very low [27]. This may result in metabolically overloaded muscle cells. Therefore, also long-term, low activity level exertions can lead to muscle fatigue, and eventually MSC.

Surprisingly, within the group of participants with ULA we did not observe a difference between test results of those with and without MSC. Possibly, these individuals developed MSC, because tissue loads and metabolic demands did not adapt adequately to the higher levels of repeated or sustained physical activity. Hence, in these individuals physical demands trespass the capacity to repair sustained damages to the musculoskeletal tissues. Consequently, normal FCE-OH test results are found, as well as a high risk on or presence of MSC. The participants who were recruited for this study, were aware of the physical demands of the study, and expressed the wish to participate, despite the possible presence of MSC. These individuals might have considered MSC to be a normal aspect of ULA [2]. In fact, many models explaining musculoskeletal disorders underline the importance of taking into account the combination of biopsychosocial factors instead of only looking at physical or work-related factors [28].

### Strength and Weaknesses

The main aim of this study was to pilot test the FCE-OH, and therefore a relatively small number of participants were included. Hence, it is not possible to make a definitive statement about the functional capacity, and the relationship between functional capacity and MSC of individuals with ULA based on this research; further research with a larger sample is needed. However, this study helped to develop hypotheses regarding functional capacity and development of MSC in individuals with ULA, and gives guidance for future research regarding this topic. Psychometric properties of general FCEs [29–31] and the FCE for individuals with work-related upper limb disorders [13] have been established. However, psychometric properties, such as safety, reliability and validity of the FCE-OH will need to be established separately, before it can be used with confidence in clinical practice. Moreover, a study is warranted, where individuals with ULA and controls are matched for work demands to allow well founded statements about the functional capacity of individuals with ULA compared to two-handed individuals with equal physical work demands. A weakness of this study is the small amount of women included. A weakness of the FCE-OH itself is that it is not possible to examine the added value of a prosthesis to the functional capacity of individuals with AE-ULA. Although a prosthesis may improve general functionality, it is not an aim of the FCE-OH to test prosthesis handling skill. Therefore, it is recommended to include a separate assessment of prosthesis handling skills when suitability of a job is examined.

## Conclusion

The FCE-OH appeared to be a feasible test to assess functional capacity in one-handed individuals. New for FCE testing is the role of a helping device, being the prosthesis. Pilot testing suggests that absolute capacity of the upper limb musculature is similar for one-handed and two-handed individuals. No relationships between test results and MSC were observed.

## Abbreviations

AE: Above-elbow (transhumeral)
BE: Below-elbow (transradial or wrist articulation)
DF: Degrees of freedom
FCE: Functional capacity evaluation
FCE-OH: Functional capacity evaluation-one-handed
MSC: Musculoskeletal complaints
NRS: Numeric rating scale
PDI: Pain disability index (questionnaire)
ULA: Upper limb absence
